# Elevated level of plasma endothelin-1 in patients with atrial septal defect

**DOI:** 10.1186/1476-7120-12-31

**Published:** 2014-08-06

**Authors:** Monika Komar, Jakub Podolec, Wojciech Płazak, Jakub Stępniewski, Bartosz Sobień, Lidia Tomkiewicz-Pająk, Tadeusz Przewłocki, Piotr Podolec

**Affiliations:** 1 Department of Cardiac and Vascular Diseases, John Paul II Hospital, Institute of Cardiology, Jagiellonian University, Str. Prądnicka 80, 31-202 Krakow, Poland; 2 Department of Haemodynamics and Angiocardiography, John Paul II Hospital, Institute of Cardiology, Jagiellonian University Medical College, Krakow, Poland

**Keywords:** Atrial septal defect, Endothelin, Congenital heart defect, Percutaneous closure

## Abstract

**Background:**

The study aimed to assess the level of plasma Endothelin-1 (ET-1) in patients before and after transcatheter closure of atrial septal defect (ASD) and to evaluate the usefulness of measuring ET-1 levels for the diagnosis and selection of candidates for ASD closure.

**Methods:**

80 patients (55 F, 25 M), mean age 42,2 ± 11,5 years were enrolled for an attempt at ASD closure. A group of 19 healthy volunteers, (12 F, 7 M) mean age 39.2 ± 9.15 served as controls. All ASD patients underwent: clinical and echocardiographic study and cardiopulmonary exercise test. ET-1 levels were measured before and after closure. Whole blood was collected from femoral artery and vein and from pulmonary artery during cardiac catheterization.

**Results:**

ET-1 levels at peripheral artery and vein in ASD patients were significantly higher than in the volunteers (p < 0.0001). The ASD subjects with highest ET-1 level presented the larger area of right ventricle and right atrium and higher pulmonary artery systolic pressure(p < 0.05). The ASD subjects with lower ET-1 level demonstrated longer time of exercise and higher peak oxygen consumption (p < 0.05). There was a decrease of ET-1 at peripheral artery (5.128 ± 8.8 vs. 2.22 ± 6.2; p < 0.001) and at peripheral vein (4.401 ± 3.33 vs. 2.05 ± 1.35; p < 0.001) within 48 hours after ASD closure, as compared to the baseline data. After 6 and 12 months farther drop in ET-1 level was observed.

**Conclusions:**

1. The level of ET-1 in ASD patients is elevated in compare to healthy subject.

2. The significant reduction of ET-1 level is observed after percutaneous closure of ASD.

3. Elevated level of ET-1 in patients with ASD is associated with right heart enlargement.

4. Measurements of ET-1 may be a supplemental diagnostic tool and may be helpful in establishing indications for defect closure.

## Background

Closure of an atrial septal defect (ASD) in patients with hemodynamically significant shunt has become standard of care in recent years. Correction of ASD prevents the development of pulmonary hypertension, cardiac arrhythmia and heart failure [1-4]. The indications for ASD closure in adults however are ambiguous. The most controversial issue is selection of candidates for ASD closure who have normal pulmonary artery pressure, absent or negligible clinical symptoms and are over 40 years of age [5-9].

In light of divergent opinions regarding ASD correction in all patients irrespective of age and clinical symptoms it appears necessary to look for novel diagnostic and prognostic indicators that may become useful for proper selection of candidates for ASD closure.

Endothelins (ET) comprise a family of three isopeptides: endothelin-1, -2, and -3. ET-1 is released mainly from endothelial cells and cardiomyocytes and is probably the most important isoform in the regulation of cardiovascular function [10,11].

ET-1 is thought to play an important role in the pathogenesis of pulmonary hypertension, both primary and secondary [12-14]. Jia B et al. [14] demonstrated elevated plasma ET-1 concentration in children with ventricular and atrial septal defects that correlated with pulmonary artery pressure. After surgical repair of the defects, plasma ET-1 concentration decreased significantly. In patients with ASD and associated elevated pulmonary artery pressure it seems important to measure ET-1 levels before and after ASD closure, to investigate the potential benefits of ET-1 receptor blockers and to establish the prognostic value of ET-1.

### Aim of the study

•to assess the level of plasma Endothelin-1 in patients with atrial septal defect

•to evaluate the usefulness of measuring ET-1 levels for the diagnosis and selection of candidates for ASD closure

## Material and methods

The study included 80 consecutive adult patients, 55 women (68.75%) and 25 men (31.25%), who underwent percutaneous closure of ASD with an Amplatzer device in the Department of Hemodynamics and Angiography, Institute of Cardiology, Jagiellonian University Medical College. The mean age of the patients was 42.2 ± 11.5 (range 18-65).

A group of 19 healthy volunteers, 12 women (61.2%) and 7 men (36.8%) with a mean age of 39.2 ± 9.15 (range 18-51), matched for age and gender served as controls.

The secundum atrial septal defect (ASD II) was diagnosed on the basis of clinical examination and transthoracic echocardiography (TTE) whereas transesophageal echocardiography (TEE) was performed to select candidates for percutaneous ASD closure. Diagnosis was confirmed during cardiac catheterization immediately before the Amplatzer deployment. The following parameters were measured:

•pulmonary artery pressure at systole (PAPs),

•total pulmonary vascular resistance (TPVR),

•pulmonary blood flow (Qp),

•systemic blood flow (Qs),

•pulmonary/systemic blood flow ratio (Qp/Qs).

Patients were considered for transcatheter closure if they had a single secundum atrial septal defect with a diameter measuring less than 30 mm on echocardiography with a rim of tissue of at least 5 mm surrounding the defect located in the central part of the septum and with a hemodynamically significant left-to-right shunt (Qp/Qs > 1.5:1). Two patients had two defects that were so close to each other that it was possible to close them with one device.

All patients underwent preoperative diagnostic procedures:

•Clinical examination including assessment of NYHA class

•Transthoracic echocardiography to measure right ventricular area at diastole (RV_area_ [cm^2^]) and right atrial area at diastole (RA_area_)

•Spirometry at rest to measure forced vital capacity expressed as a percentage of the normal value (FVC%), forced expiratory volume in one second expressed as a percentage of the normal value (FEV_1_%)

•Exercise spirometry test to measure duration of exercise in seconds (T), peak oxygen consumption VO_2peak_(ml/kg/min), time to anaerobic threshold (T_AT_), oxygen consumption at the anaerobic threshold (VO_2AT_%), ventilatory equivalent for carbon dioxide (VE/VCO_2_)

### Measurement of endothelin levels

Endothelin-1 levels were measured immediately before and at 2 days, 6 and 12 months after transcatheter closure.

Whole blood was collected from femoral artery and femoral vein in each ASD II patient before the procedure and in volunteers. Additionally, blood samples were collected from pulmonary artery during diagnostic cardiac catheterization. After transcatheter closure blood was sampled from femoral artery and femoral vein.

Whole blood was collected in precooled tubes containing EDTA and aprotinine (1 vol of anticoagulant for 9 vol of blood) and immediately placed on ice. The blood was then centrifuged to the freezing temperature at 3000 rpm for 10 min. The platelet-poor plasma was frozen in tubes and sent to the laboratory. Measurements of ET-1 were done at the Biochemical Laboratory of the John Paul II Hospital in Krakow. ET-1 was measured with a commercial immunoenzymatic ELISA method and results expressed as fmol/ml. The study protocol was approved by the Bioethics Committee of the Jagiellonian University Medical College (KBET/262/B/2002).

Analysis was performed in the whole group and in two subgroups with the high and low plasma ET-1 levels in the pulmonary artery:

•Subgroup G – ET-1 levels from 0.91 to 2.81 fmol/ml

•Subgroup H – ET-1 levels from 7.13 to 24.68 fmol/ml

**Figure 1:**
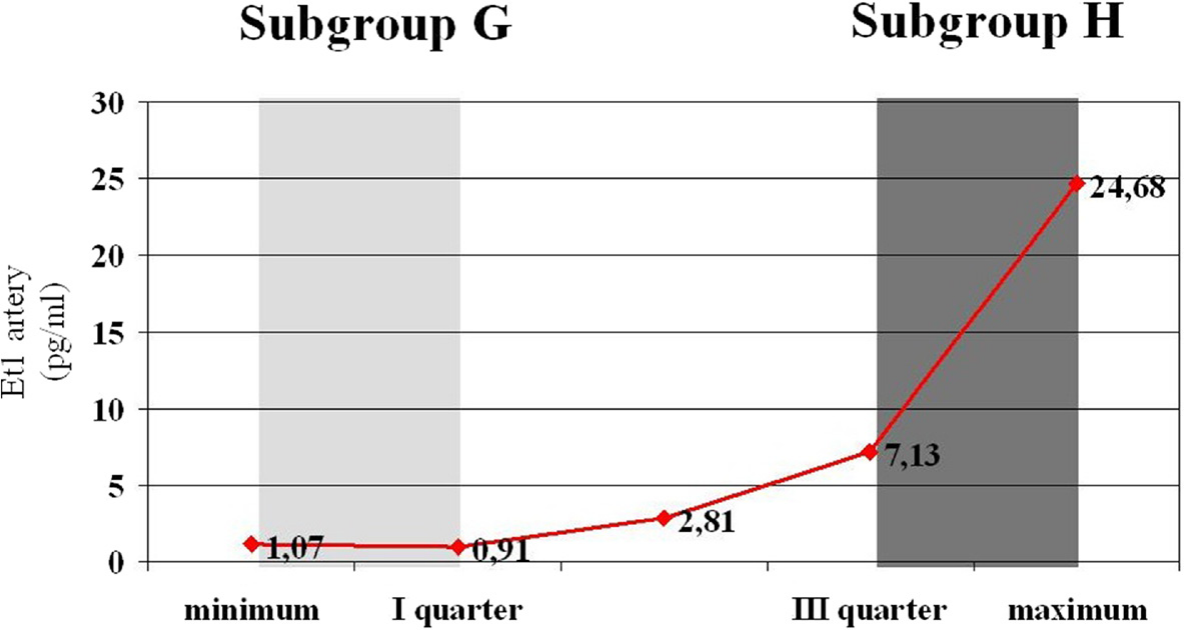
Plasma ET-1 levels in the pulmonary artery in patients with ASD II before transcatheter procedure – sorted by quartiles (gray vertical bars denote the first and fourth quartile).

Patient characteristics in subgroup G and H are summarized in Table 1.To allocate patients to the subgroups ET-1 values were sorted in ascending order to define the median and quartiles. Those who had ET-1 levels in the first quartile were allocated to subgroup G and those with ET-1 levels in the fourth quartile to subgroup H (Figure 1).

**Table 1:** Patient characteristics in subgroup G and H with the low and high ET01 levels, respectively

	**Subgroup G n = 30**	**Subgroup H n = 38**	**p**
ET-1 (fmol/ml)	1.81 ± 1,3 (0.91-2.81)	9.4 ± 9.2 (7.72-24.68)	**<0,0001**
Women	20 (66.7%)	25 (65.9%)	**NS**
Men	10 (33.3%)	13 (34.1%)	**NS**

### Statistical analysis

The discrimination value of selected parameters was determined by comparing two subgroups with the low and high ET-1 levels and ROC analysis was performed to define the cut-off values and calculate sensitivity and specificity. The Peto method was then used to perform meta-analysis ad calculate odds ratios (OR) for individual risk factors. The Peto method also allowed for assessment of multidimensional risk of elevated ET-1 levels. OR were calculated with corresponding 95% confidence intervals (95% CI).

Multivariate analyses such as multiple forward stepwise regression, logistic regression and canonical correlation were used to evaluate the parameters affecting ET-1 levels. Stepwise and logistic regression allowed for estimation of the effect of independent variables on the dependent variable i.e. endothelin levels. Statistical significance was set at α ≤0.05. Statistical analyses were performed using Statistica 6.0. Meta-analysis and ROC analysis were performed using StatsDirect 2.1.

## Results

An Amplatzer device was implanted without major complications in all eligible patients. Minor complications during transcatheter closure included transient supraventricular tachycardia in 5 patients (6.25%) and transient bradycardia to 30 bpm in one patient (1.25%). 5 patients (6.25%) had a hematoma at the femoral puncture site. The mean duration of the procedure including diagnostic right-heart catheterization was 41.0 ± 7.2 (20-65) min and the mean fluoroscopy time 12.2 ± 4.42 (5-27) min. The size of Amplatzer devices ranged from 13 to 40 mm (mean 24.2 ± 6.9 mm). Immediately after deployment of the device TEE revealed nonsignificant residual shunt in 8 patients (10%) persisting 2 days after the procedure TTE and resolving at 6 months of follow-up in each case.

### ET-1 levels before and after transcatheter closure

Table 2 summarizes ET-1 levels in patients before and after transcatheter closure of ASD II and in healthy volunteers.

**Table 2:** ET-1 levels in patients before transcatheter closure of ASD II and in healthy volunteers

**Group**	**Vessel**	**Mean**	**Minimum**	**Maximum**	**SD**	***p *** **ASD II vs. healthy**
**ASD II patients before closure n = 80**	**Peripheral artery**	6,122	0,772	13,981	3,651	< 0,0001
	**Peripheral vein**	4,431	1,230	13,546	3,312	< 0,0001
	**Pulmonary artery**	5,541	0,91	24,68	6,874	-
**Healthy volunteers n = 19**	**Peripheral artery**	0,049	0,010	0,098	0,025	< 0,0001
	**Peripheral vein**	0,054	0,001	0,099	0,027	< 0,0001

**Figure 2:**
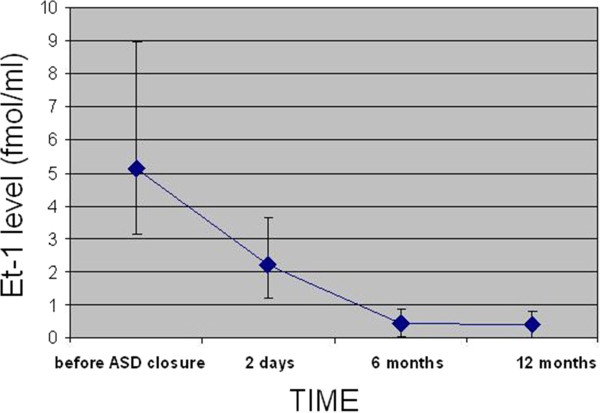
ET-1 levels in the peripheral artery before and after transcatheter closure of ASD II.

**Figure 3:**
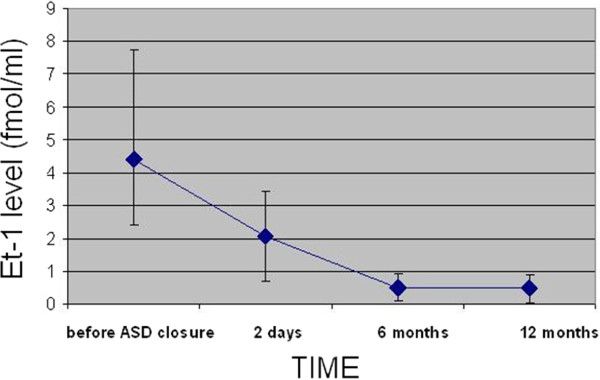
ET-1 levels in the peripheral vein before and after transcatheter closure of ASD II.

Patients before transcatheter closure of ASD II had significantly higher ET-1 levels both in peripheral artery and in peripheral vein as compared with healthy volunteers (p < 0.0001). The minimum detectable level of ET-1 in the peripheral artery and vein in ASD patients was higher than the maximum level in healthy volunteers. The highest level of ET-1 was detected in the pulmonary artery in ASD patients (mean 5.541 ± 6.87).After transcatheter closure ET-1 levels significantly decreased both in peripheral artery and vein in all patients. Figures 2 and 3 depict ET-1 levels in the peripheral artery and vein before and after transcatheter closure of ASD II.

As early as 48 hours after ASD II closure the level of ET-1 was significantly reduced both in the peripheral artery (2.10 ± 6.7; p < 0.001) and vein (2.05 ± 1.15; p < 0.001). At 6 months the levels of ET-1 were further reduced to a mean of 0.49 ± 0.17 in the peripheral artery and to 0.40 ± 0.19 in the peripheral vein. At 12 months there was no further significant decrease as compared with the levels at 6 months.

At 12 months after transcatheter closure there was still a significant difference in ET-1 levels both in the peripheral artery (0.049 ± 0.025 vs. 0.49 ± 0.17, p < 0.001) and in the peripheral vein (0.054 ± 0.027 vs. 0.40 ± 0.19, p < 0.001) between patients with closed ASD and healthy volunteers.

Selected parameters, including hemodynamics and physical capacity, were compared in two subgroups with the high (subgroup H - ET-1 levels from 7,13 to 24,68 fmol/ml) and low (subgroup G – ET-1 levels from 0,91 to 2.81 fmol/ml) ET-1 levels in the pulmonary artery.

Patients in subgroup H were significantly older than those in subgroup G.

ASD II patients with the high ET-1 level had significantly increased right atrial and right ventricular area and higher pulmonary artery pressures.

ASD II patients with the low ET-1 level had higher peak oxygen consumption and at the anaerobic threshold (p < 0.05) and time to anaerobic threshold was significantly prolonged (p < 0.01). The ventilatory equivalent for carbon dioxide was significantly lower in subgroup G. Table 3 summarizes the results of the comparative analysis.

**Table 3:** Selected parameters in patients with the high (subgroup H) and low (subgroup G) ET-1 levels in the pulmonary artery

	**Subgroup G – ET1 from 0,91 to 2,81 fmol/ml**	**Subgroup H – ET1 from 7,13 to 24,68 fmol/ml**	***P***
**Age**	35,1 ± 11,43	56,36 ± 5,7	**<0,001**
**Defect size**	23,1 ± 7,28	18,9 ± 4,28	NS
**RV**_**area **_**(cm**^**2**^**)**	23,1 ± 1,01	25 ± 1,51	**< 0,01**
**RA**_**area **_**(cm**^**2**^**)**	16,2 ± 1,2	19,5 ± 0,9	**<0,001**
**PAPs (mmHg)**	20,1 ± 5,2	40,01 ± 19,5	**<0,01**
**Qp (l/min)**	8,4 ± 2,41	8,7 ± 2,5	NS
**COP (dyn/s/cm**^**−5**^**)**	140,1 ± 24,8	189 ± 130,1	NS
**Qp/Qs**	2,20 ± 1,31	2,34 ± 1,4	NS
**T (s)**	734 ± 71,45	700,7 ± 90,1	NS
**VO**_**2peak**_**(ml/kg/min)**	24,13 ± 3,32	21,1 ± 3,92	**<0,05**
**FVC%**	107,6 ± 14,0	89,5 ± 4,7	**<0,05**
**FEV**_**1**_**%**	93,5 ± 10,2	87,3 ± 17	NS
**T**_**AT **_**(s)**	455,6 ± 44,9	390 ± 23,1	**<0,01**
**VO**_**2AT**_**%**	41,4 ± 2,8	35,41 ± 4,5	**<0,05**
**VE/VCO**_**2**_	30,4 ± 3,1	39 ± 4,4	**<0,05**
**NYHA**	1,9 ± 1,08	2,39 ± 1,03	NS

The canonical correlation analysis showed that the following parameters had an influence on ET-1 levels in pulmonary artery, peripheral artery and vein: PAPs, Qp/Qs, age, RV_area_ (cm^2^), RA_area_ (cm^2^), size of ASD II, VO_2peak_ (ml/kg/min), FVC (%), VO_2AT_%; p < 0.05.

The multiple forward stepwise regression analysis revealed that of all parameters [(PAPs, Qp/Qs, age, RV_area_ (cm^2^), RA_area_ (cm^2^), size of ASD II, VO_2peak_ (ml/kg/min), FVC (%), VO_2AT_%, T_AT_)] PAPs (F(7.25) = 9.1785; p < 0.0001, standard error 2.170) had the strongest influence on ET-1 levels.

**Figure 4:**
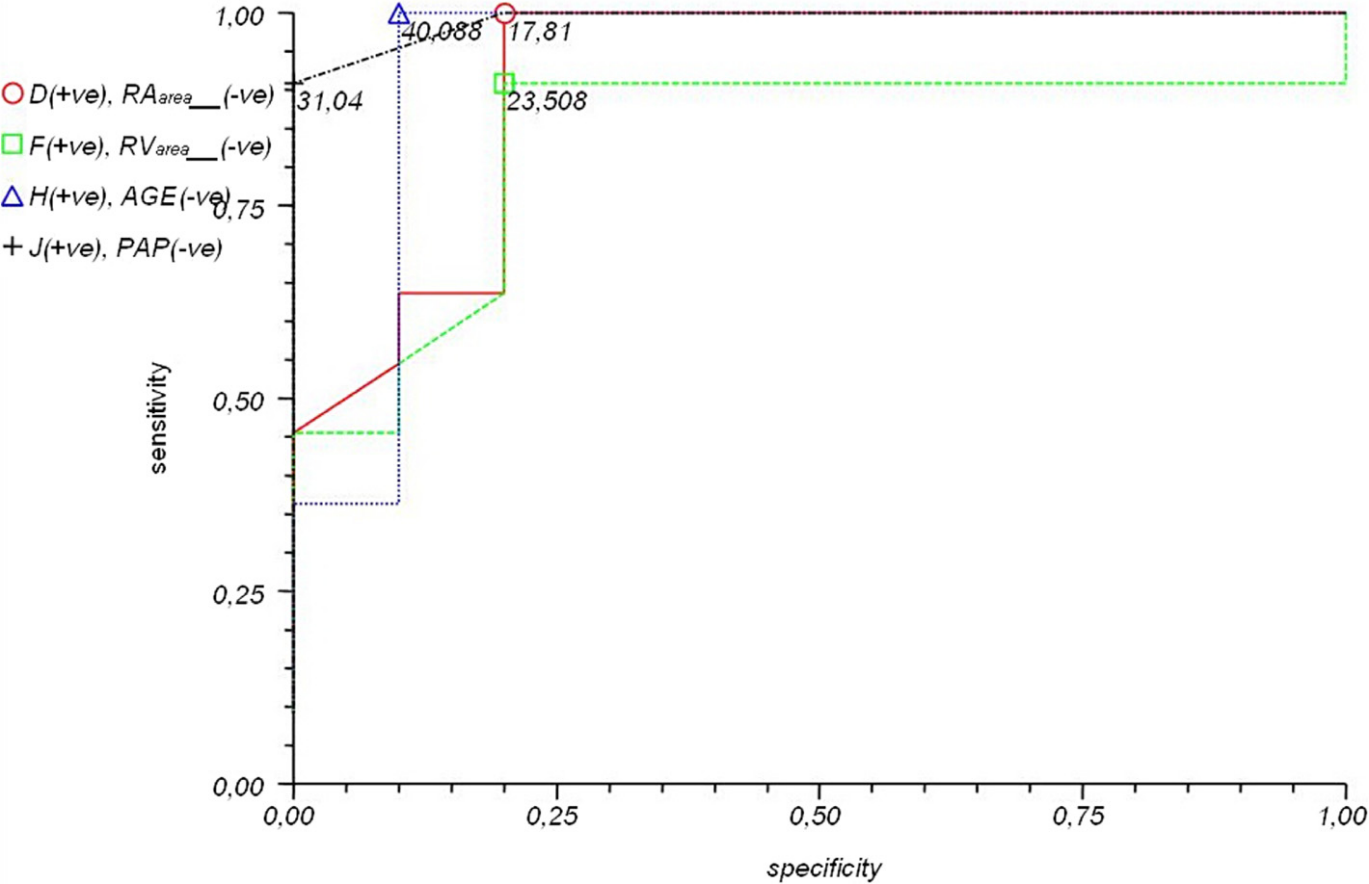
**ROC curves for selected parameters: [RV**_**area **_**(cm**^**2**^**) – green, RA**_**area **_**(cm**^**2**^**) – red, PAPs (mmHg) – black and age – blue] and cut-off values.**

**Figure 5:**
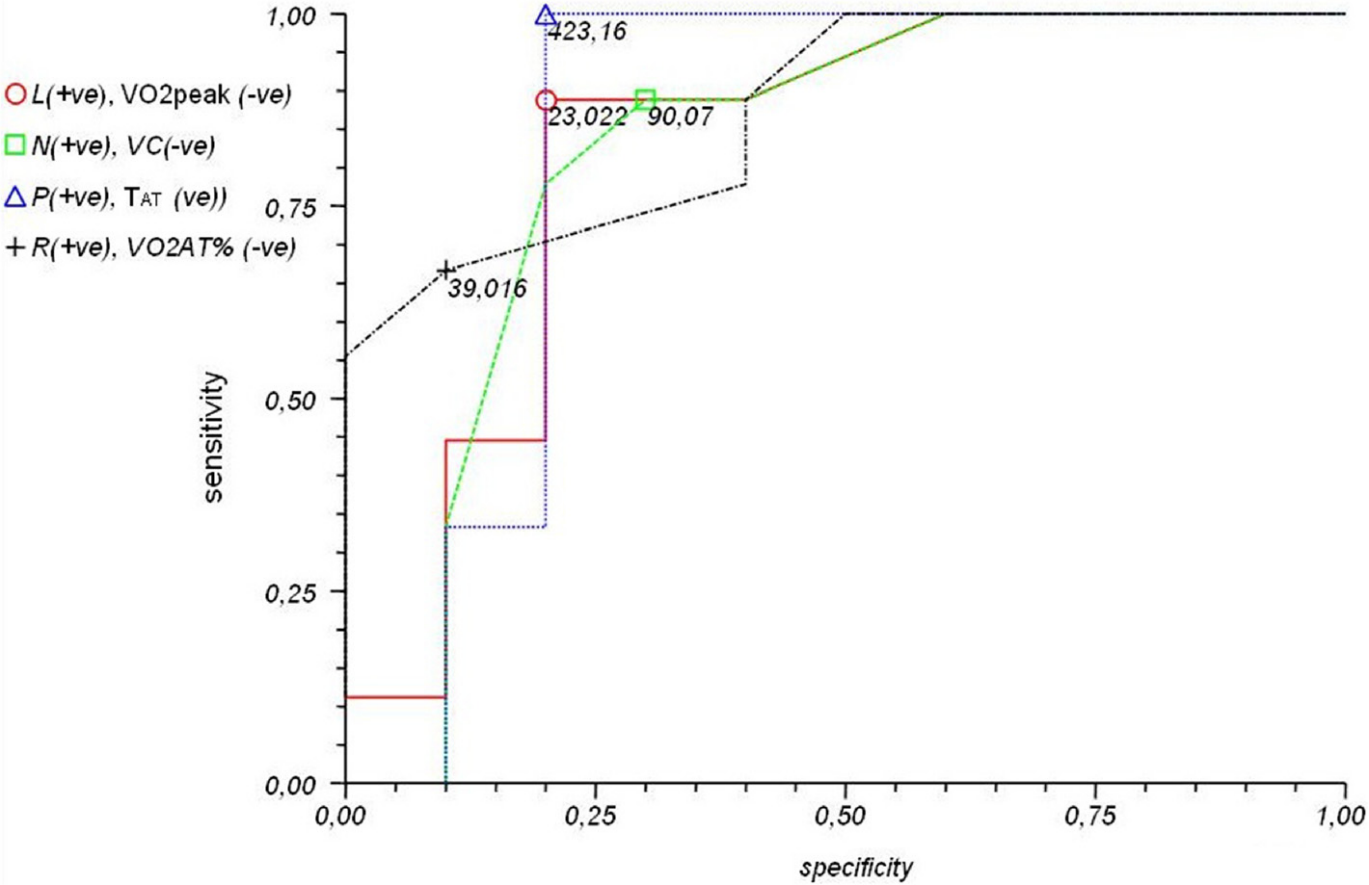
**ROC curves for selected parameters [VO**_**2peak**_**(ml/kg/min) – red, FVC (%) – green, VO**_**2AT**_**% – black, T**_**AT **_**– blue] and cut-off values.**

**Figure 6:**
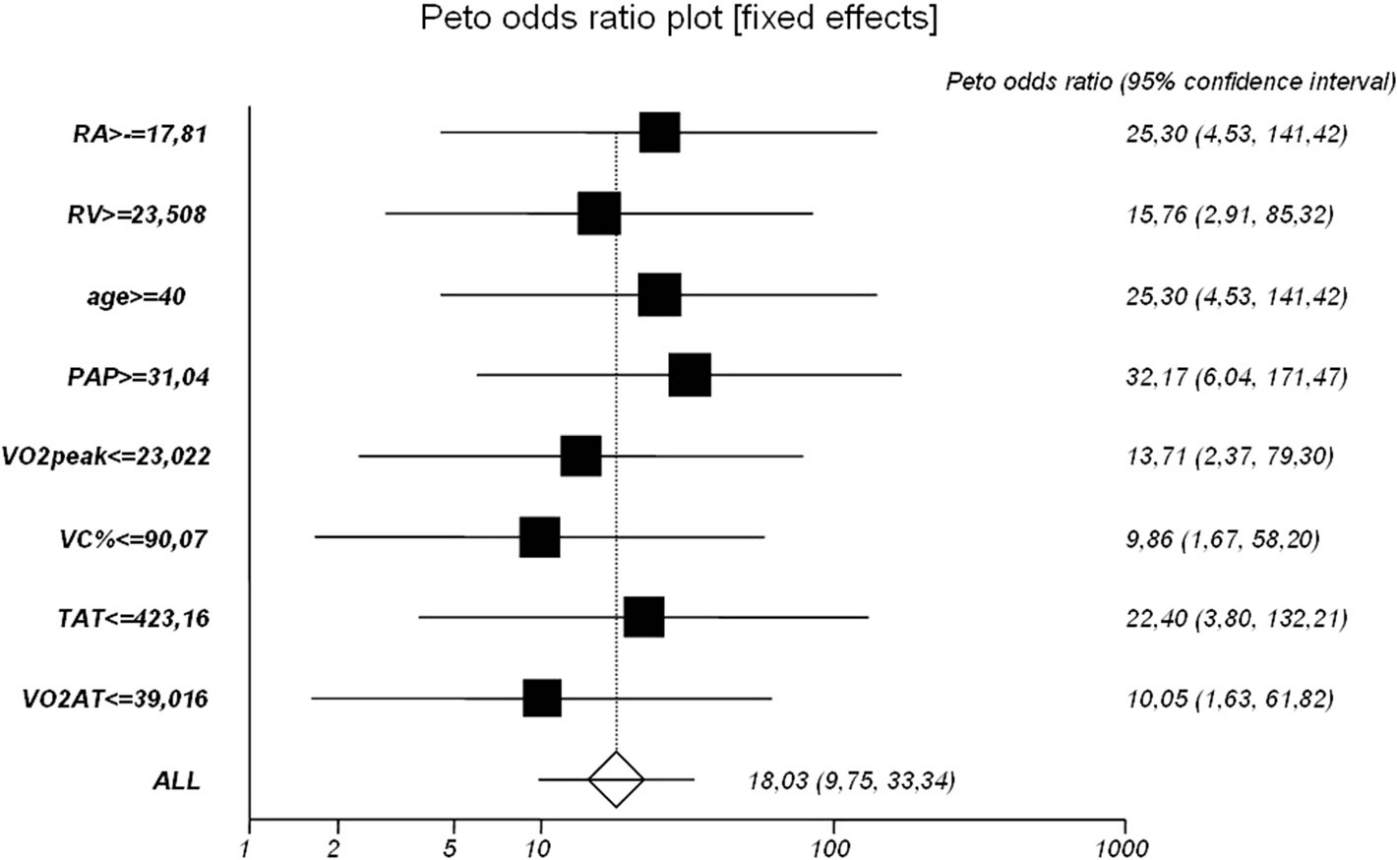
**Meta-analysis of selected parameters influencing elevated ET-1 levels using the Peto method.** Odds ratios (OR) with corresponding 95% confidence intervals (95% CI).

Higher ET-1 levels before closure don’t predict higher pulmonary artery pressure/resistances after closure.ROC curves for selected parameters influencing ET-1 levels and meta-analysis using the Peto method are depicted in Figures 4, 5 and 6.

The cut-off values defined in the meta-analysis and increasing the risk of elevated ET-1 levels in ASD II patients are as follows:

•Right atrial area (RAarea) > 17.81 mm (p < 0.0002)

•Right ventricular area (RVarea) > 23.508 mm (p < 0.0014)

•Age > 40 years (p < 0.0002)

•Pulmonary artery pressure at systole (PAPs) > 31.04 mmHg (p < 0.0001)

•Peak oxygen consumption (VO_2peak_) < 23.022 ml/kg/min (p < 0.0002)

•Forced vital capacity (FVC) < 90.07% (p < 0.0115)

•Time to anaerobic threshold (TAT) < 423.16 second (p < 0.0006)

•Oxygen consumption at the anaerobic threshold (VO_2AT_%) < 39.016%VO2 (p < 0.0128)

## Discussion

Percutaneous closure of interatrial septal defects has become a standard therapeutic approach in the last few years. Correction of the defect may prevents the development of pulmonary hypertension, rhythm disturbances or failure of the heart. Amplatzer Septal Occluders have been commonly used for defect closure since the 90s [1,3,5,6].

Elevated circulating ET-1 is observed in patients with left-to-right shunt and pulmonary hypertension indicating that pulmonary vessels are responsible for increased ET-1 synthesis [14].

Endothelin is probably one of the key contributors to the pathogenesis of pulmonary hypertension [12-14].

In the present study ASD II patients before transcatheter closure had significantly higher ET-1 levels both in peripheral artery and vein as compared with healthy volunteers. The minimum detectable ET-1 in the peripheral artery and vein in ASD II patients was higher than the maximum ET-1 level in healthy volunteers. The highest ET-1 level was detected in the pulmonary artery in ASD II patients. The multiple forward stepwise regression analysis revealed that pulmonary artery pressure had the strongest influence on ET-1 levels. These findings are concordant with the results obtained by other investigators [15-19]. The high circulating ET-1 as compared with healthy volunteers is accounted for by increased pulmonary blood flow, increased pulmonary pressure and increased synthesis of ET-1 [13-19].

ET-1 levels decreased significantly both in the peripheral artery and the peripheral vein in all patients as early as 2 days after transcatheter closure. Jia et al. [14] obtained similar results.

In the present study ET-1 levels further decreased at 6 and 12 months, although the rate of decrease was much slower after 6 months. The decrease in ET-1 concentration as early as at 2 days after ASD closure confirms it is a volume response which depends on a significant reduction in pulmonary blood flow. In the present study selected parameters, including hemodynamics and physical capacity, were compared in two subgroups of patients with the high and low ET-1 levels. The high ET-1 levels were correlated with age, right atrial and ventricular enlargement and elevated pulmonary artery pressure. Patients with ASD II and high ET-1 had lower peak oxygen consumption and at the anaerobic threshold and shorter time to anaerobic threshold corresponding to reduced physical capacity. Patients with low ET-1 were classified as those in the first quartile (<2.99 pg/ml) whereas patients with high ET-1 as those in the fourth quartile (>6.99 pg/ml). In the present study we defined the cut-off values of ET-1 above which we can expect reduced physical capacity and right atrial and right ventricular enlargement. Measurements of ET-1 in ASD patients with borderline shunt ratio may help identify the subjects with elevated pulmonary artery pressure who may benefit from ASD closure.

The significant ASD, according to the ESC Guidelines, is defined as shunt with signs of right ventricular volume overload despite of Qp:Qs. Also patients who are asymptomatic or mildly symptomatic should be offered defect closure, because the natural course of untreated atrial septal defects often leads to a shortened life expectancy compared with healthy subjects [20,21]. Measurements of ET-1 in peripheral blood may be a useful tool for diagnosis and selection of patients with borderline left-to-right shunt ratio for suitable intervention. Thus we could propose transcatheter closure in patients with ASD with insignificant shunt and high ET-1 levels. Conversely we could suggest a medical follow-up (versus closure) in patients with small ASDs with insignificant shunt and low ET-1 levels. It seems appropriate to consider ET-1 dosage in different populations with congenital heart disease, where the decision or the timing for intervention is still under debate.

## Conclusions

1. The level of plasma Endothelin-1 in patients with atrial septal defect is elevated in compare to healthy subject

2. Elevated level of Endothelin-1 in patients with ASD is associated with right heart enlargement and poor exercise capacity.

3. After percutaneus closure of secundum ASD using ASO device the significant reduction of ET level is observed.

4. Measurements of ET-1 in peripheral blood and possibly in pulmonary artery may be a supplemental diagnostic tool and a prognostic indicator and may be helpful in establishing indications for defect closure.

## Abbreviations

ASD: Atrial septal defect; ET-1: Endothelin-1; FVC%: Forced vital capacity expressed as a percentage of the normal value; FEV1%: Forced expiratory volume in one second expressed as a percentage of the normal value; OR: Odds ratios; PAPs: Pulmonary artery pressure at systole; Qp: Pulmonary blood flow; Qs: Systemic blood flow; Qp/Qs: Pulmonary/systemic blood flow ratio; RVarea: Right ventricular area at diastole [cm2]; RAarea: Right atrial area at diastole; T: Duration of exercise in seconds; TAT: Time to anaerobic threshold; TEE: Transesophageal echocardiography; TPVR: Total pulmonary vascular resistance; TTE: Transthoracic echocardiography; VO2AT%: Oxygen consumption at the anaerobic threshold; VE/VCO2: Ventilatory equivalent for carbon dioxide.

## Competing interests

The authors declare that they have no competing interests.

## Authors’ contributions

MK: contributed to study conception and design, acquisition of data, analysis and interpretation of data, drafting the manuscript, revising it critically for important intellectual content. JP: contributed to study conception and design, helped to draft the manuscript, revising it critically. WP: contributed to study conception and design, revising the draft critically for important intellectual content, gave final approval of the version to be published. JS: analysis and interpretation of data, drafting the manuscript. BS: contributed to study conception and design, acquisition of data, analysis and interpretation of data, drafting the manuscript. LTP: contributed in acquisition of data, analysis and interpretation of data, drafting the manuscript, revising it critically for important intellectual content. TP: contributed to study conception and design, acquisition of data, analysis and interpretation of data, drafting the manuscript, revising it critically for important intellectual content, gave final approval of the version to be published. PP: contributed to study conception and design, revising it critically for important intellectual content, gave final approval of the version to be published. All authors read and approved the final manuscript.
